# Comprehensive micropollutant screening using LC-HRMS/MS at three riverbank filtration sites to assess natural attenuation and potential implications for human health

**DOI:** 10.1016/j.wroa.2018.100007

**Published:** 2018-11-02

**Authors:** Juliane Hollender, Judith Rothardt, Dirk Radny, Martin Loos, Jannis Epting, Peter Huggenberger, Paul Borer, Heinz Singer

**Affiliations:** a Eawag, Swiss Federal Institute of Aquatic Science and Technology, Ueberlandstrasse 133, 8600 Duebendorf, Switzerland; b Applied and Environmental Geology, University of Basel, Bernoullistrasse 32, 4056 Basel, Switzerland; c Institute of Biogeochemistry and Pollutant Dynamics, Universitätstrasse 16, ETH Zürich, 8092 Zurich, Switzerland

**Keywords:** Groundwater, LC-HRMS/MS, Riverbank filtration, Drinking water, Organic contaminants, Risk assessment

## Abstract

Riverbank filtration (RBF) is used worldwide to produce high quality drinking water. With river water often contaminated by micropollutants (MPs) from various sources, this study addresses the occurrence and fate of such MPs at three different RBF sites with oxic alluvial sediments and short travel times to the drinking water well down to hours. A broad range of MPs with various physico-chemical properties were analysed with detection limits in the low ng L^−1^ range using solid phase extraction followed by liquid chromatography coupled to tandem high resolution mass spectrometry. Out of the 526 MPs targeted, a total of 123 different MPs were detected above the limit of quantification at the three different RBF sites. Of the 75–96 MPs detected in each river, 43–59% were attenuated during RBF. The remaining total concentrations of the MPs in the raw drinking water accounted to 0.6–1.6 μgL^−1^ with only a few compounds exceeding 0.1 μgL^−1^, an often used threshold value. The attenuation was most pronounced in the first meters of infiltration with a full elimination of 17 compounds at all three sites. However, a mixing with groundwater related to regional groundwater flow complicated the characterisation of natural attenuation potentials along the transects. Additional non-target screening at one site revealed similar trends for further non-target components. Overall, a risk assessment of the target and estimated non-target compound concentrations finally indicated during the sampling period no health risk of the drinking water according to current guidelines. Our results demonstrate that monitoring of contamination sources within a catchment and the affected water quality remains important in such vulnerable systems with partially short residence times.

## Highlights

• 123 of 500 micropollutants were detected with LC-ESI-HRMS/MS in surface and groundwater samples.
• About 50% of the micropollutants were attenuated during riverbank filtration with short residence times.
• 17 micropollutants were attenuated >50% at all 3 sites within <24 h and 13 persisted >50%.
• Target and non-target screening of raw water indicate no significant human health risk.

## Graphical abstract

**Figure:**
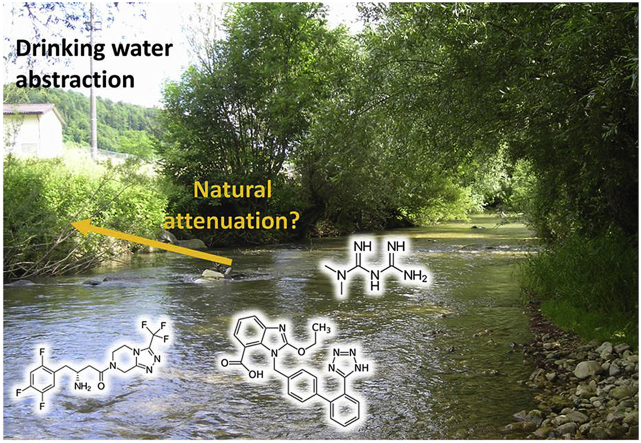

## 1 Introduction

Riverbank filtration (RBF) is used worldwide as a natural process to produce high quality drinking water with relatively low environmental impact and at low capital costs (Maeng et al., 2011). Due to discharges of wastewater treatment plants (WWTPs), as well as urban and agricultural runoff, river water contains nutrients, pathogens, and a multitude of organic micropollutants (MPs), such as pharmaceuticals, pesticides, biocides, illicit drugs, personal care products, and industrial chemicals, especially in densely populated areas (Daughton and Ternes, 1999; Heberer, 2002; Perez and Barcelo, 2007). Although the vast majority of MPs are present in the low ng per liter range, many of these compounds can nevertheless raise environmental and human health issues (Burkhardt-Holm, 2010; Jones et al., 2001; Kummerer, 2009; Schwarzenbach et al., 2006). The infiltration of such surface water might lead to a consequent transfer of MPs into groundwater used for drinking water extraction. Hence, the load of MPs in surface water pose a potential risk to water supplies (Barber et al., 2013; Kunkel and Radke, 2012) and pose a considerable risk for ensuring drinking water quality criteria.

Varying MP loads discharged with WWTP effluents due to differences in regional usage and prescription, as well as varying WWTP efficiencies implicate observable stream-to-stream and within-stream spatio-temporal variability of MP concentrations. Processes including biotransformation, photolysis, sorption, dispersion, and volatilization influence the fate and attenuation of MPs in aquatic systems (Escher and Fenner, 2011; Gurr and Reinhard, 2006; Schwarzenbach et al., 2006). All these processes are affected by the physico-chemical properties of the compounds as well as the physical and biological parameters of the river, such as river flow rate, turbidity, dissolved oxygen concentration, pH, temperature, the structure of microbial communities, the hydraulic regime and the extent of hyporheic exchange in the river (Boulton et al., 2010). In addition, mixing with groundwater that is not influenced by surface water may considerably complicate the identification of natural attenuation potentials. Several studies strongly indicate that natural attenuation is most pronounced already in the first few meters of infiltration, i.e., within the highly reactive hyporheic zone (Brunke and Gonser, 1997; Heberer et al., 2008; Huntscha et al., 2013; Lawrence et al., 2013; Regnery et al., 2015).

Despite its importance, few attempts have been made to correlate this attenuation potential of MPs with their physico-chemical properties. Polar compounds with low log *K*_*OW*_ (octanol water distribution coefficient) values which are negatively charged or neutral move rapidly with the infiltrating river water into the groundwater and can only be eliminated by microbial degradation (Huntscha et al., 2013). In contrast, neutral compounds with higher log *K*_ow_ values or positively charged compounds can also adsorb to organic matter which is usually slightly negatively charged (Benotti et al., 2012; Ramil et al., 2010). Cationic compounds can additionally sorb to clay surfaces (Droge and Goss, 2013). As mentioned above, these general trends are modulated by many parameters such as temperature, discharge, organic carbon content, and the residence time in the riverbank (Massmann et al., 2006; Rauch-Williams et al., 2010; Storck et al., 2012). Recently, organic compounds that have substantial persistence (freshwater half-life >40 days according to REACH definition) and mobility characteristics (log D_oc_ (organic carbon water distribution coefficient) < 4.5 between pH 4–10) to be transported through natural and urban barriers and reach sources of drinking water have been defined as PMOCs (persistent and mobile organic chemicals) and have received more attention (Arp et al., 2017).

In general, information on vertical and lateral transport of MPs within surface waters and from surface water into aquifers is scarce. Only few studies have been conducted to evaluate the natural attenuation potential of selected MPs in different aquifer types. For example, the attenuation potential of a rapidly recharging karst aquifer in southern Germany with short residence times was investigated using waste-water specific tracers (Hillebrand et al. 2012, 2015). High attenuation rates with degradation half-lives ranging from 37 h to 90 h were obtained for atenolol, caffeine, ibuprofen, and paracetamol, depicting the high degradability of these compounds. The fate of MPs in the hyporheic zone and the influence of temperature and redox potential on attenuation was also studied at an eutrophic lowland stream (Lewandowski et al., 2011) and a sediment core in the laboratory (Burke et al., 2014). Except for benzotriazole and carbamazepine remaining persistent, degradation rates decreased from warm to cold and oxic to manganese reducing conditions in the laboratory experiments. In another bench scaled flume experiment designed to study hyporheic processes (Li et al., 2015), transformation products (TPs) were detected which were only formed in the sediment but released to the water, hinting at secondary contamination with TPs (Writer et al., 2013). Kunkel and Radke (2012) provided a list of favourable attenuation conditions within rivers located in Central Europe and reported higher attenuation for sunny, dry weather periods during periods of low discharge.

In comparison to the above mentioned studies, in Switzerland mostly aerobic conditions prevail in the aquifers, and groundwater usually contains less dissolved organic carbon (DOC). Hence, oxic, redox-independent conditions predominate RBF processes. As a consequence of the predominance of mainly alluvial gravel aquifers, RBF is often highly dynamic with short groundwater residence times. The growing number of river restoration projects that are conducted along Swiss rivers as modern flood protection measures and to achieve a “good ecological status” of rivers as required by the European Water Framework Directive (European Parliament, 2000) might further decrease the average residence times in RBF systems by removal of bank fixation and widening of the river (Schirmer et al., 2014). Swiss legislation for drinking water abstraction requires only a minimum subsurface residence time of 10 days, 5 times less than in the European Union, and about 30% of Swiss drinking water is produced at RBF sites. The resulting raw water extracted at RBF sites is frequently only marginally treated to account for bacterial contamination (e.g., by UV lamps). An upgrade of those treatment chains to abate MPs would be quite costly since many small decentral drinking water production sites are spread over Switzerland.

Therefore, an improved understanding of the environmental fate and transport of MPs during RBF is essential to provide a basis on which to decide whether drinking water sources are sufficiently protected or additional measures are needed. As thousands of MPs in daily use enter rivers directly or indirectly after incomplete treatment from their various sources such as agriculture, households and industry, it is extremely important to study not only a few selected compounds herein, but to get a comprehensive overview on MPs in surface waters that either attenuate or persist during RBF.

Thus, in the presented study we aimed to determine the occurrence of several hundreds of MPs from various sources at three different RBF sites with varying residence times from hours to months to get a comprehensive picture on the contamination. More than 500 MPs with a broad range of physico-chemical properties were analysed with detection limits in the low ng L^−1^ range using solid phase extraction followed by liquid chromatography coupled to high resolution tandem mass spectrometry (LC-HRMS/MS). The concentrations of MPs in each river sample and the respective fate during RBF was evaluated, correlated with physico-chemical data and compared with literature data. Additionally, a profiling of peak pattern of non-target compounds was conducted at one site. Finally, the implications with regard to drinking water consumption of the resulting raw water was assessed. Due to the breadth of compounds it was not possible to investigate the time-variable nature of contaminant distribution in the river and groundwater samples in more detail and constrain fate processes.

## 2 Materials and methods

### 2.1 Field sites

**Fig. 1:**
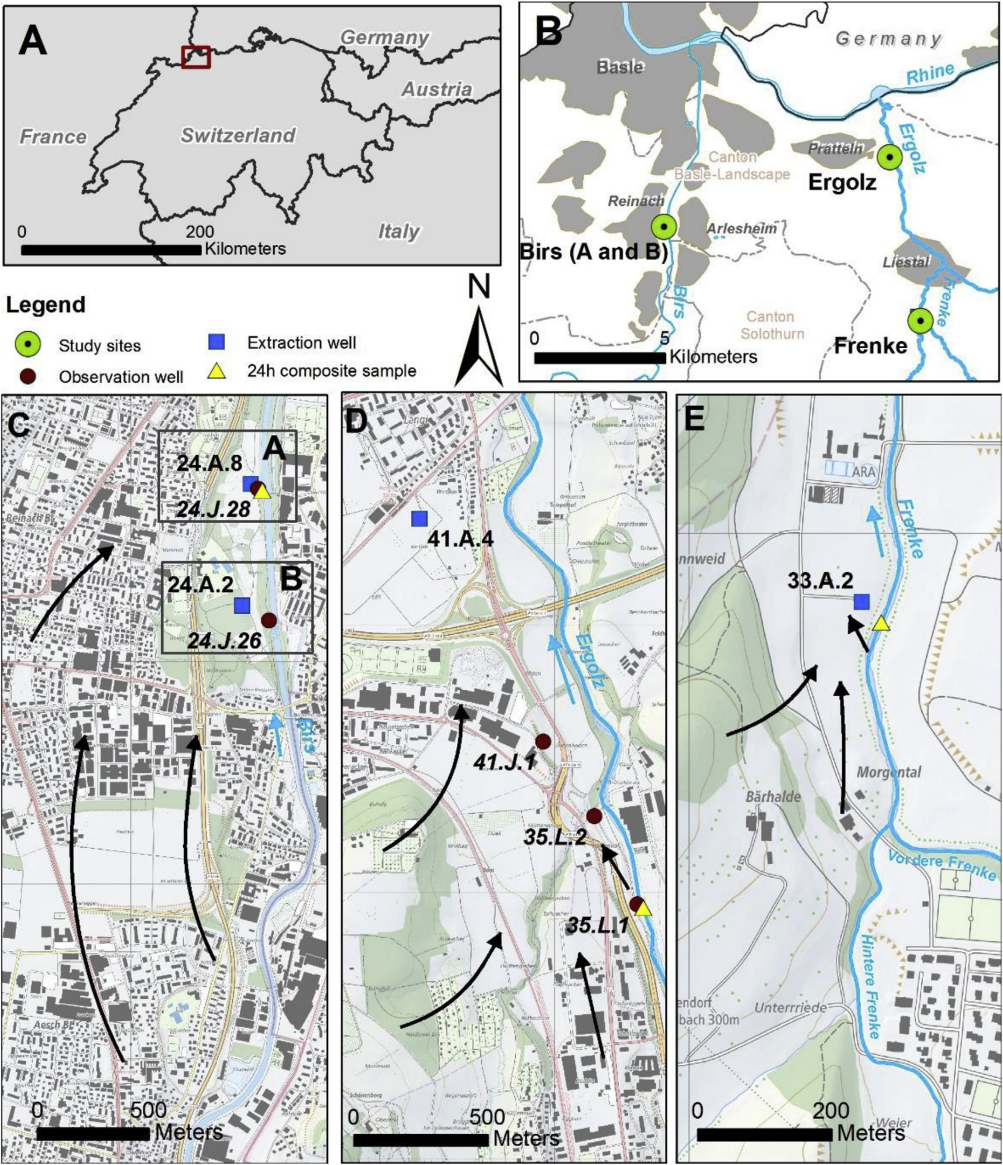
**A and B)** Location of the study sites within Switzerland (A) and in the canton Basle-Landscape (B); Detailed view of transects with the respective wells: **C**) Birs, **D**) Ergolz; **E**) Frenke; black arrows indicate both the local (bank filtration) and the regional groundwater flow direction; blue arrows indicate the flow direction of the rivers. Data source base maps: Esri data/AND Data Solutions, B.V. (subfigures A and B); swisstopo, Swiss Map Raster, Bundesamt für Landestopographie (Art.30 Geo IV): 5704 000 000, reproduced by permission of swisstopo/JA100119 (subfigures C to E). (For interpretation of the references to colour in this figure legend, the reader is referred to the Web version of this article.)

The study was conducted at three different RBF sites along the rivers Birs, Ergolz and Frenke, all located in the north-western part of Switzerland, in the canton of Basle-Landscape (Fig. 1). For a detailed description of the RBF sites including the geological and hydrogeological settings please refer to (Epting et al., 2018). Along the three river sites no lakes or reservoirs are located upstream; together with the geology and topography within the catchment area this results in abrupt discharge changes after heavy rainfall events, implying highly dynamic river-systems. All three locations are characterized by alluvial aquifer systems. The aquifer material mainly consists of carbonate gravel (Triassic and Jurassic) composed by well-rounded, variably sorted sediments with few layers of clay and silt in between, resulting in a large variance in hydraulic properties, whereas hydraulic conductivities range from 10^−5^ to 10^−2^ m s^−1^. The rivers were once canalized at the end of the nineteenth century, and river bed levels have subsequently eroded by several meters into the former floodplains, from which they were eventually disconnected. Whereas the rivers at the study sites Birs and Frenke are connected to the groundwater systems, at the location of the study site Ergolz the river is partly disconnected from the groundwater system and infiltration occurs via the unsaturated zone (Epting et al., 2018).

Discharges recorded between 1917 and 2013 at the river Birs (BAFU discharge station 2106 “Muenchenstein, Hofmatt”) ranged from 0.83 to 383 m^3^ s^−1^and at the Ergolz (BAFU discharge station 2202 “Liestal”) from 0.1 to 146 m^3^ s^−1^, respectively. Discharge at the Frenke (Vordere Frenke, Bubendorf) was 0.04–27.3 m^3^ s^−1^ between 1978 and 2013 (Amt für Umweltschutz und Energie, 2013).

The land use within the whole region of the Canton Basle-Landscape is dominated by agricultural (41%) and forestal use (41%), and a smaller portion by urban areas (18%), historically most pronounced close to the rivers. In total, 30 WWTPs with a population equivalent of over 220000 are connected to the different streams in the Canton, resulting in a proportion of up to 13%, 23%, and 7% treated wastewater during base flow conditions in the rivers Birs (Q_95%_: 3.1 m^3^/s), Ergolz (Q_95%_: 0.5 m^3^ s^−1^), and Frenke (Q_95%_: 0.2 m^3^ s^−1^), respectively (Amt für Umweltschutz und Energie, 2015).

At the three sampling sites in total four RBF transects were investigated (Table 1). Transect Birs A included the observation well 24.J.28, and the extraction well 24.A.8, which was out of operation during the sampling campaign (Fig. 1C). Transect Birs B included the observation well 24.J.26, and the extraction well 24.A.2 (extraction rate approximately 0.04 m^3^ s^−1^ during sampling). Both transects were chosen according to previous investigations from Affolter et al. (2010), documenting an intense river-groundwater interaction at these sites with short groundwater residence times (less than 20 h from the river to the observation wells 24.J.26 and 24.J.28). Maximal groundwater depth at the Birs site is around 29 m and the thickness of the saturated zone varies between 0.6 and 10 m due to the bedrock topography as well as the transition zone of the bedrock to the hill slope to the West. Like at all other investigated transects at the rivers Ergolz and Frenke (Table 1), the redox groundwater conditions at the Birs are oxic (Page et al., 2012).

**Table 1:** Characterisation of the sampling from surface water and groundwater along the transects (location see Fig. 1); no travel times are shown for the wells 24.A.8, 24.A.2. (both Birs site), 41.J.1 and 41.A.4 (both Ergolz site) as those wells are also impacted by the regional groundwater flow, that superposes signals from bankfiltration. ^1^ from experimental data; ^2^ based on Darcy's law including the water levels measured at the day of sampling; T, temperature; EC, electric conductivity; DOC, dissolved organic carbon.

Measuring point	Site	Date	T [°C]	pH	EC [μS cm^−1^]	Redox [mV]	O_2_ [mg L^−1^]	DOC [mg CL^−1^]	Mean travel time [h]	Discharge [m^3^ s^−1^]
24 h river sample	Birs	2.-3.12.2013	8.3	8.3	506	250	12.1	1.6	0	12.7
24.J.28	Birs A	03.12.2013	10.5	7.1	510	203	8.5	1.1	10–14^1^	–
24.A.8	Birs A	03.12.2013	13.1	7.3	519	227	7.8	0.6		–
24.J.26	Birs B	03.12.2013	8.0	7.7	493	241	7.2	1.4	19–21^1^	–
24.A.2	Birs B	03.12.2013	11.5	7.5	483	216	5.7	0.7	–	–

24 h river sample	Ergolz	4.-5.12.2013	6.5	8.4	667	177	12.4	1.7	0	3.0
35.L.1	Ergolz	05.12.2013	6.4	8.0	667	166	9.5	1.5	14^1^	–
35.L.2	Ergolz	05.12.2013	9.0	7.9	652	120	10.2	1.4	39^1^	–
41.J.1	Ergolz	05.12.2013	13.1	7.6	641	124	8.0	1.4	>300^2^	–
41.A.4	Ergolz	05.12.2013	11.8	7.4	705	123	6.5	0.9	>700^2^	–

24 h river sample	Frenke	4.-5.12.2013	6.9	8.6	627	234	11.9	1.8	0	0.4
33.A.2	Frenke	05.12.2013	11.2	7.3	637	257	6.8	1.2	16^1^	–

The Ergolz transect includes three observation wells: 35.L.1, 35.L.2, and 41.J.1, and the extraction well 41.A.4, which is extracting approximately 0.08 m^3^ s^−1^, mainly during night time. Whilst the observation wells 35.L.1 and 35.L.2 are close to the river bank (10 m and 45 m, respectively), the distance between 41.J.1 and the river is around 280 m and the extraction well 41.A.4 is located around 1500 m downgradient of the river sampling site (Fig. 1D). With this setup, the Ergolz transect is different to the other two sites. However, as highest attenuation rates can be observed close to the riverbank, the results from the observation wells 35.L.1 and 35.L.2 are representing the attenuation capacity at the Ergolz site. For these two observation wells, travel times were calculated before the sampling campaign by conducting a non-parametric deconvolution of time series of the electrical conductivity (EC) (Cirpka et al., 2007; Vogt et al., 2010), measured both in the river and at the two wells (Table 1) with loggers from Sensor Technik Sirnach (type DLN70) from February 2014 till September 2014.

For the quantitative MP screening at the Frenke-transect only data from the river Frenke and the extraction well 33.A.2 were included as the extraction well is very close to the riverbank (around 40 m perpendicular to the Frenke river, Fig. 1E) with mean travel times during the campaign of less than 20 h (Table 1). In general, the extraction well is operated during night time with an extraction rate of around 0.04 m^3^ s^−1^.

The organic carbon content in the fine material of the aquifer material at the sites of Frenke and Ergolz ranged from 0.1 to 1% (method see SI). With a typical particle size contribution of the material <0.2 mm of 2–5% determined for the Frenke sample, the fraction of organic carbon f_oc_ of the whole aquifer material at the investigated sites can be assumed to range in between 0.002 and 0.05%. This is in the same order found for fluvial sandy gravels in the prealpine Thur valley aquifer in Switzerland (Huntscha et al., 2013).

### 2.2 Sampling

All samples were taken during low discharge conditions in December 2013 when the concentrations in rivers are expected to be relatively stable and wastewater-derived compounds usually show the highest concentrations (see Table 1 for exact discharge values during the sampling campaign). The sampling of 24-h composite river samples at each river site was performed with automated samplers placed on a flat riverbank, where 150 mlL samples were sampled hourly in a 9.5 L glass bottle (Teledyne Isco 6712 sampler, Lincoln). Groundwater and raw drinking water grab samples were sampled in 5 different observation wells at all four transects, as well as at four drinking water extraction wells, taking the retention time in the riverbank into account (Table 1).

The samples at the observation wells were taken by submersible electric pumps (Whale pumps, type: Gp1352) with an average pumping rate of 10 L min^−1^. In the well houses of the extraction wells the samples were taken directly from the tap. In all cases, the physico-chemical parameters temperature, pH, specific electrical conductivity (EC), the oxidation-reduction-potential (ORP) as well as dissolved oxygen concentrations were measured (HACH-LANGE, HQ40d). Sample collection was conducted after stabilization of the physico-chemical parameters which was achieved after around 10–15 min.

Even though air temperatures were below 5 °C during the sampling campaign, all samples obtained in the field were immediately placed in a cool box equipped with thermal packs. All samples obtained during a day were transported to the laboratory and stored at −4 °C in the dark until sample preparation and analyses.

### 2.3 Chemicals

Ammonia (25%), ammonium acetate, formic acid (98–100%), and ethanol (HPLC purity) were obtained from Merck AG. Methanol (MeOH, Optima^®^ LC/MS 99.9%) was purchased from Thermo Scientific, and ethyl acetate (EtOAc, HPLC purity) from Sigma Aldrich. Ultra purity water was obtained using a NANOpure^®^21 Barnstead from Thermo Fisher Scientific. High-purity N_2_ (99.99995%) was purchased from Carbagas.

In total 526 reference compounds (425 parent compounds, and 101 major metabolites, see SI Table S7, Fig. S1) were included in the analytical method. Isotopically labelled internal standards and unlabelled reference standards originated mainly from Toronto Research Chemicals, Dr. Ehrenstorfer GmbH, Sigma-Aldrich and a few from Lipomed AG.

### 2.4 Sample preparation

After thawing, the raw water samples were filtered through a glass microfiber filter (GF/F, 47 mm, pore size 0.7 μm, Whatman) by vacuum filtration to remove any particulate matter. Before solid phase extraction (SPE), ammonium-acetate buffer was added (1 M, 1 mL) and the pH was adjusted to 6.5 using formic acid and ammonia. The exact volume of 1 L was spiked with 163 isotope labelled internal standards (see SI Table S1) before processing of the sample. Additionally, at least one blank sample, a field blank, and a calibration curve were prepared and processed in the same way. Separately, for three samples (namely Birs, and Ergolz 24-h composite samples, and well house sample 41.A.4) standard addition was performed previously to sample enrichment to obtain limits of quantification (LOQ) and relative recoveries, especially for those compounds for which no own labelled internal standard was available.

For the enrichment a SPE-LC-HRMS/MS method as described by Moschet et al. (2013) with slight modifications was used. Briefly, filtered water samples (1 L) were concentrated by SPE on a 12-fold vacuum extraction box (Visiprep, 12 ports, Sigma Aldrich) using in-house filled three-layered cartridges (polypropylene, 6 ml, Carl Roth GmbH) of Oasis HLB (200 mg, Water AG), a mixed-bed layer of Strata-X CW/AW/ENV+ (1:1:1.5, 350 mg, Phenomenex) and a third layer of ENVI-Carb (200 mg, Sigma-Aldrich). The different layers were separated with matching frits (polyethylene, 20 μm, Carl Roth GmbH). The SPE cartridges were consecutively conditioned with 5 mL methanol, and 10 mL H_2_O. Water samples were passed through the cartridges with a consistent speed of 3–5  mL min^−1^. Afterwards the loaded cartridges were dried completely on the vacuum manifest by pulling air through the cartridge and stored overnight at 4 °C until elution. The cartridges were eluted in back-flush mode using ethylacetate/methanol (1:1, 6 ml) containing ammonium (0.5%), and subsequently ethylacetate/methanol (1:1, 3 mL) containing formic acid (1.7%). Afterwards the cartridges were rinsed with pure methanol (2 mL) resulting in a final combined neutral extracts of 11 mL. Sample aliquots were reduced to 100 μL under a gentle nitrogen gas stream at 35 °C. The extracts were reconstituted with 900 μL of Milli-Q water and centrifuged for 30 min at 35′000 rpm using a Heraeus Megafuge 1.0R (Thermo Scientific).

### 2.5 LC-HRMS/MS analysis

Sample analysis was performed on a high performance liquid chromatograph (HPLC) coupled to a tandem high-resolution mass spectrometer (HR-MS/MS). The HPLC consisted of a CTC Pal auto sampler (CTC analytics), a RHEOS 2200 pump with degasser (Flux Instruments) and a column oven. The analytes were separated using a reversed phase column (XBridge™ C18 column, 3.5 μm, 2.1 × 50 mm, Waters) coupled with a pre-column (3.5 μm, 2.1 × 10 mm, same material). The mobile phase consisted of water and methanol both acidified with formic acid (0.1%) and a gradient from 10 to 95% methanol was applied. The injection volume was 20 μL, and the mobile phase flow was 0.2 mL min^−1^.

Detection of analytes was performed using a Q-Exactive™ Hybrid Quadrupole-Orbitrap mass spectrometer (ThermoFisher Scientific), with an electrospray ionization (ESI) probe performed in two separate runs for the positive and negative ionization modes each. Full scan accurate mass spectra were acquired from 100 to 1000 m/z with a nominal resolving power of 70′000 referenced at m/z 200. The automated gain control (AGC) was set to 500′000 and the maximal injection time was 200 ms with a mass accuracy of ±5 ppm. Data-independent high-resolution product ion spectra (HR-MS/MS) were recorded at a resolving power of 17′500 at m/z 200, AGC set to 200′000 and maximal injection time to 100 ms. Quantification was carried out with a calibration row of extracted standards between 0.5 and 750 ng L^−1^ using Tracefinder 3.1. (ThermoFisher Scientific). For substances without own internal standard the closest-matching internal standard according to retention time and structure was used. These substances were corrected for relative recovery using spiked control samples. Limits of quantification (LOQs) were determined based on the external calibration curve (lowest standard with peak and signal-to-noise above 10) and corrected for matrix effects using river water (Birs) and groundwater (Ergolz).

### 2.6 Characterisation of unknown peaks at Ergolz site

The samples from the Ergolz transect (except for the sample from the observation well 41.J.1) were re-measured in triplicate in the positive ionization mode on a Q Exactive Plus Orbitrap with an increased nominal resolution of 280′000 at m/z 200 (Thermo Fisher Scientific). The raw files were converted with ProteoWizard v 3.0.7162 from raw to mzXML formats (Holman et al., 2014). Further data analysis was performed in the R statistical environment (R Team, Accessed 01 January 2016) using the enviMass (Loos, Accessed 22 August 2016) software workflow. With this workflow, extracted ion chromatograms were first detected, a peak picking run and non-replicable peaks discarded (parameters given in Table S1). For all remaining peaks per replicate the masses were then recalibrated (parameters given in Table S2), their intensities normalized and a retention-time dependent limit of detection estimated. All isotopic peaks of target and internal standard compounds were removed for major adducts [M + H, M + NH_4_, M + Na, M + K] (Table S3) to focus on non-target peaks only. Thereupon, a blank peak subtraction was conducted (with and without internal standards), based on a 100-fold intensity threshold (Table S4). Subsequently, a non-targeted adduct- [M + H, M + NH_4_, M + Na, M + K] and resolution-specific isotopologue-grouping (Table S5) was carried out for all peaks in each sample. Finally, intensity changes of a mass over the sites and replicates were extracted using an intensity descent over all pooled peaks, resulting in so-called non-target profiles (Table S6). Profiles of different adducts and isotopologues of the same non-target analyte were further combined to components based on the intensity correlation among all profiles of similar retention time. The most intense profiles per component were then used with tailored R scripts to derive Venn diagrams (Hanbo, Accessed 22 August 2016) and cumulative intensity distributions. Moreover, concentrations for these non-target profiles were estimated combining calibration models of 43 randomly selected target internal standard/standard pairs.

## 3 Results and discussion

### 3.1 Micropollutants (MPs) selection and coverage by analytical method

526 target compounds were selected based on consumption data and occurrence in former monitoring campaigns of surface and groundwater in Switzerland (Huntscha et al., 2012; Moschet et al., 2014; Schymanski et al., 2014) and covered pharmaceuticals (215), plant protection products (abbreviated in the following as pesticides, 142), biocides (18), illicit drugs (11), perfluorinated compounds (PFCs, 16), others (23 including 8 food additives and 15 industrial compounds), as well as 101 major metabolites (mostly from pesticides and pharmaceuticals) for which reference standards were available (Table S7, Fig. S1). The compounds covered a broad range of physico-chemical properties with log *K*_*OW*_: 3–9, pH dependent octanol-water distribution coefficient log *D*_*ow*_: −5.7–6.9 (at pH 6.6–6.9), molecular size: 101–1447 Da, different speciation, and molecular structures containing various functional groups (Fig. S2, predictions using Episuite (US EPA, 2012)).

Due to the enrichment by various mechanisms using five different solid phase materials, for more than 70% of the compounds relative recoveries in the range of 80–120% were achieved (Fig. S3). Together with an enrichment factor of 1000, and the sensitive and selective detection by HRMS/MS, from the overall 526 MPs included in the analytical method for 80% the LOQ was below 10 ng L^−1^ and for 24% even below 1 ng L^−1^ in the surface and groundwater samples. The determined LOQs varied only slightly between the surface and groundwater (Fig. S4).

### 3.2 MPs pattern in surface water

**Fig. 2:**
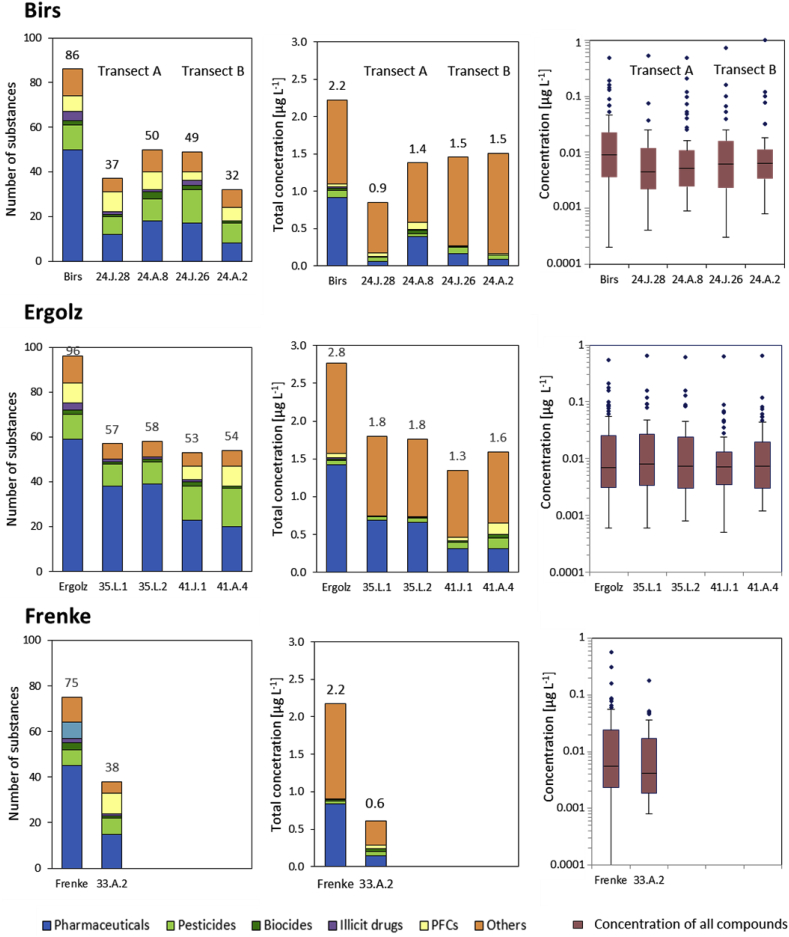
Overview of the number of detected compounds and the total concentration at the three sites Birs, Ergolz and Frenke sorted according to substance classes, as well as boxplots of the concentrations with median, the first and third quartile, whiskers representing the standard deviations, and outliers. Individual values are provided in Table S7.

Out of the 526 MPs that could be quantified, a total of 123 were detected above the LOQ at the three different RBF sites (individual values in Table S7). Therefrom, in total 86, 96, and 75 were detected in the three different 24-h composite samples of the rivers Birs, Ergolz, and Frenke, respectively (Fig. 2). Median concentrations of all quantified MPs were 9.0, 6.9, and 5.6 ng L^−1^ in these rivers, respectively (Fig. 2). The highest quantified concentration in the 24-h composite river water samples were the persistent artificial sweeteners acesulfam and sucralose, the anti-corrosive agent benzotriazole, the drugs metformin, cetirizine and paracetamol as well as caffeine. These findings are in line with other surface water monitoring studies in Europe and Switzerland (Huntscha et al., 2012; Loos et al., 2009). However, the concentrations were below 1 μg L^−1^ for individual compounds and apart from the above mentioned below 0.1 μg L^−1^, in agreement with the wastewater proportion (Amt für Umweltschutz und Energie, 2015). The WWTPs upstream the RBF sites exhibit obviously sufficient elimination efficiencies as well-degradable compounds such as paracetamol or mefenamic acid, which are used in high amounts in Switzerland, were only detected in relatively low concentrations or even below their LOQs. These concentrations may even be lower at other times, since our sampling was conducted in winter when slightly lower degradation in the wastewater treatment plant can be expected (Munz et al., 2017). Hence, the quantified concentrations of the MPs in the three rivers are distinctly lower compared to previously published studies from other countries where either higher portions of treated wastewater were usually reported in the rivers or less treatment or even sewage overflow of untreated wastewater occurred (Acuna et al., 2015; Bradley et al., 2014; Hughes et al., 2013; Regnery et al., 2015; Writer et al., 2013). As our primary focus was on the elucidation of contamination from wastewater, we sampled under dry weather conditions, so that neither storm water overflow nor run-off from agricultural fields was anticipated. Similarly, and with the sampling period ranging outside the respective application period, we also did not expect pesticides peak concentrations to occur. However, as recently observed (Munz et al., 2017), pesticides can be detected all over the year due to biocide use as well as accidental spills. Nevertheless, only between 7 and 12 pesticides or pesticide TPs from the 207 included in the analysis were detected, with concentrations below 0.03 μg L^−1^. This might even result predominantly from exfiltration of groundwater upstream of the RBF sites such as the TPs from chloridazon, metolachlor, and atrazine (application outphased in Switzerland). Even though the highest number of compounds detected was in the river Ergolz, where also the highest proportion of treated wastewater within the three different sites was reported (up to 23%), the highest median concentrations were determined for the river Birs (Fig. 2). However, differences between number of compounds and median concentrations were small and the total concentration was rather driven by a few compounds with higher concentrations. Overall, the observed substance spectrum and the quantified concentrations did not vary strongly at the different locations. Between 45 (Frenke) and 59 (Ergolz) pharmaceuticals were detected, overall 70 different pharmaceuticals from 251 analysed.

### 3.3 MPs attenuation during riverbank filtration at individual sites

In all observation well samples collected a few meters (≤20 m) from the river with low residence times in the aquifer the number of quantified MPs > LOQ decreased to 43% and 56% (Birs, tansect A and B), 59% (Ergolz), and 51% (Frenke), respectively, showing overall a good RBF potential. At the Birs transect A the number of quantified MPs decreased from 86 in the river to 37 at the observation well 24.J.28 within 10–14 h flow time, which could also be observed in the significant decline of the median concentration from 9.0 to 4.4 ng L^−1^ (Fig. 2). However, an increase of quantified MPs was documented for the well house sample 24.A.8 (50 MPs with a median concentration of 5.2 ng L^−1^). According to Epting et al., (2018), for this RBF site groundwater at the extraction well 24.A.8 consisted of up to 80% of regional groundwater during the sampling time of early December 2013, and was only marginally influenced by infiltrating river water. Thus, the increase in the number of detects can likely be attributed to other urban contamination sources within the contributing area. At the Birs transect B, the number of quantifiable MPs was reduced within 19–21 h mean travel time to the observation well 24.J.26 from 86 to 49 and to the extraction well 24.A.2 further to 32. The median concentration dropped from 8.8 to 6.2 ng L^−1^ in the first observation well and afterwards remained almost constant.

A comparable situation could also be documented for all the samples obtained at the Ergolz site. Again the number of detected MPs decreased markedly from 96 to 57 in the first observation well and then remained constant within the measurement uncertainty - taking into account that quite some compounds were quantified close to the LOQ (Fig. 2). Similarly, the total concentration of MPs decreased significantly from 2.8 to 1.8 μg L^−1^ but the differences between the median concentrations are rather marginal.

The investigations performed at the Frenke transect did not include a groundwater observation well (Fig. 2), but the extraction well is located closely to the river with a calculated residence time of only 16 h (calculation based on Darcy's law including the water levels measured at the day of sampling). Nevertheless a significant decrease in the number of detected MPs from initially 75 to 38 was documented, which goes along with a reduction in the median concentration of 5.6 to 4.2 ng L^−1^. According to (Epting et al., 2018) a typical riverbank filtration situation was captured during the low-flow sampling.

### 3.4 Generalization of MPs behaviour during riverbank filtration

As expected, at all three sites the well-known persistent compounds carbamazepine, acesulfam, and sucralose that are often used as wastewater tracers showed no clear decreasing trend from surface to groundwater samples (Benotti et al., 2012; Bradley et al., 2014; Huntscha et al., 2012; Scheurer et al., 2011; Van Stempvoort et al., 2011; Zirlewagen et al., 2016). To account for dilution often ratios between concentrations of MPs and a conservative tracer are used in lieu of actual concentrations (Huntscha et al., 2013; Scheurer et al., 2011). However, the change of the tracer concentrations compared to the surface water samples showed not the same trend for the three tracers and changed also differently along transects. This may likely be attributed to fluctuating tracer concentrations in wastewater effluents, combined with the difficulty to sample the same water package along the transect and mixing with other, regional groundwater fractions. Overall, we conclude that those tracers cannot be reliably used to correct for the dilution in such a dynamic system and consequently, all concentration changes must be interpreted with care. The first observation wells (extraction well for Frenke) with the lowest mean travel time (<24 h; Table 1) can best be linked to the 24 h-composite samples of the surface water, respectively. Consequently, we attributed only MPs with concentration decreases >50% between the surface water and the first observation well at all three sites (Frenke, Ergolz, Birs B) as natural attenuated by retardation or degradation. In addition, only compounds with concentrations higher than three times their LOQ in the surface water were included in this assessment as otherwise dilution can easily lead to misinterpretations due to concentrations falling below their LOQ. Using this approach, 17 compounds (Table 2) were consistently eliminated by natural attenuation at all three sites. On the contrary, 13 MPs (Table 2) including the three tracers mentioned above showed <50% elimination at all three sites and were hence considered persistent. The remaining 40–50 compounds at each site were either not ubiquitously detected (e.g., several pesticides, x-ray contrast media, illicit drugs, or the well degradable paracetamol), the concentrations were not three times higher than their LOQ (e.g., PFCs, the well degradable mefenamic acid), or the concentrations decrease varied between < and >50% at the sites (e.g., benzotriazole, atenolol acid) so that no unambiguous classification could be achieved.

**Table 2:** Compounds attenuated at all three sites more than 50% (top) or persisted at all three sites less than 50% (bottom) with physico-chemical properties and maximum concentration (max. conc.) in the three extraction wells. a, anionic; n, neutral; c, cationic; z, zwitterionic; Pharm, pharmaceutical; Met, metabolite.

	CAS	Compound class	log Kow^a^	Speciation at pH 7	pKa^a^	Max. conc. extraction wells [μg L^−1^]
**Natural attenuated compounds**						
4-Acetamidoantipyrin	83-15-8	Pharm Met	0.3	n	12.5	<LOQ
Atenolol	29122-68-7	Beta blocker	0.16	c	9.7	<LOQ
Bezafibrat	41859-67-0	Lipid lowering	4.25	a	3.8	0.002
Caffeine	58-08-2	Stimulant	−0.07	n	0.9	0.076
Clopidogrel carboxylic acid	144457-28-3	Pharm Met	1.51	z	7.9	<LOQ
Diclofenac	15307-86-5	Anti-inflammatory	4.02	a	4.0	<LOQ
Etodolac	41340-25-4	Anti-inflammatory	3.93	a	4.7	<LOQ
Flufenamic acid	530-78-9	Anti-inflammatory	5.25	a	3.9	<LOQ
Irbesartan	138402-11-6	Antihypertensive	5.31	a	4.1	<LOQ
Metformine	657-24-9	Antidiabetic	−2.64	c	10.3	<LOQ
Metoprolol	37350-58-6	Betablocker	1.88	c	9.7	<LOQ
Naproxen	22204-53-1	Anti-inflammatory	3.18	a	4.2	<LOQ
Saccharine	81-07-2	Sweetener	0.91	a	2.8	0.008
Sitagliptin	486460-32-6	Antidiabetic	1.39	c	8.8	<LOQ
Trimethoprim	738-70-5	Antibiotic	0.73	c	7.2	0.003
Valsartan	137862-53-4	Antihypertensive	3.65	a	4.4	<LOQ
Valsartanic acid	164265-78-5	Pharm Met	1.83	a	4.0	<LOQ

**Persistent compounds**						
Acesulfame	55589-62-3	Sweetener	−1.33	a	3.0	1.00
Atrazine-2-Hydroxy	2163-68-0	Herbicide Met^b^	2.09	n	3.0	0.010
Candesartan	139481-59-7	Antihypertensive	4.79	a	3.9	0.018
Carbamazepine	298-46-4	Anticonvulsant	2.45	n	16.0	0.041
Chloridazon-desphenyl	6339-19-1	Herbicide Met	−0.41	z	6.6	0.020
Chloridazon-methyl-desphenyl	17254-80-7	Herbicide Met	−1.37	n	15.8	0.016
2,6-Dichlorobenzamide	2008-58-4	Herbicide^b^ Met	0.9	n	12.1	0.003
Hydrochlorothiazide	58-93-5	Antihypertensive	−0.07	n	9.1	0.038
Lamotrigine	84057-84-1	Anticonvulsant	2.57	n	5.9	0.044
4/5-Methyl-benzotriazole	136-85-6	Industrial	1.71	n	8.9	0.081
Metolachlor-ESA	171118-09-5	Herbicide Met	1.69	a	13.7	0.007
Sucralose	56038-13-2	Sweetener	−1	n	11.9	0.120
Sulfamethoxazole	723-46-6	Antibiotic	0.89	n	2.0	0.020

a US EPA, 2012.

b Parent compound no longer registered in Switzerland.

For most of the compounds compiled in Table 2, sorption to organic material is expected to be negligible since log *K*_*OW*_ values are relatively low or the compounds are anionic (e.g., diclofenac, irbesartan). Additionally, the fraction of organic carbon f_oc_ of the aquifer material is estimated to be quite low (0.002%–0.05%). For the positively charged atenolol and metoprolol cation exchange was reported to be relevant (Schaffer et al., 2012). However, due to the low f_oc_ and results from a similar Swiss aquifer (Huntscha et al., 2013), such retardation is also expected to be of minor importance for those compounds.

Similar to the results in this study biodegradation has already been reported in RBF studies for compounds such as atenolol (Bradley et al., 2014; Huntscha et al., 2013), caffeine (Hillebrand et al., 2015), metformin (Bradley et al., 2014), diclofenac, 4-actamidoantipyrine, metoprolol (Huntscha et al., 2013; Burke et al., 2014), naproxen and trimethoprim (Regnery et al., 2015). For sulfamethoxazole partial degradation during oxic laboratory column tests was reported (Baumgarten et al., 2011; Bertelkamp et al., 2016a), but minor attenuation was determined at any of the four studied transects, as well as elsewhere (Benotti et al., 2012; Bradley et al., 2014; Storck et al., 2012). For benzotriazole, a slight degrading trend at all transects was observable, despite persistence documented in other studies (Burke et al., 2014; Reemtsma et al., 2010). In contrast to the so far mentioned compounds, information on the behaviour of sartans during RBF is scarce (Nödler et al., 2013). Irbesartan, valsartan and their TP valsartan acid were well degraded at all three sites whereas candesartan showed persistence despite their similar structure. However, the overall behaviour is in general agreement with the biodegradability reported in WWTPs, with candesartan showing persistence as well (Bayer et al., 2014). No information about the newer antidiabetic drug sitagliptin is available in literature. In a recent study we found persistence of sitagliptin in WWTPs (Goetz and Singer, 2015) but obviously during RBF the cationic amine can be attenuated, either by biodegradation or sorption. The persistence of the less studied hydrochlorothiazide and lamotrigine is in agreement with recent literature (Bertelkamp et al., 2016a, b, Bollmann et al., 2016). Persistence of the pesticide TPs of chloridazon, atrazine and dichlobenil is well known (Buttiglieri et al., 2009; Kern et al., 2011; Kolpin et al., 1998). For the latter two pesticides application is no longer permitted for some years already but they still can be found in groundwater as well as surface water due to exfiltration (Loos et al., 2010; Reemtsma et al., 2013).

For MPs, characterized either as natural attenuated or persistent at the three sites (Table 2), there is no clear trend between physico-chemical properties (log K_ow_, log D_ow_) and behaviour. This is complicated by the fact that sorption and degradation cannot be distinguished. With regard to biodegradability the prediction program (BIOWIN, US EPA, 2012) comprises a series of six aerobic and one anaerobic models to correlate the presence of structural fragments with biodegradation data and persistence from different databases (Howard et al., 1992; Jaworska et al., 2003). The results for the seven models (Table S8) show no correlation between the persistence of the compounds and the predictions based on structural moieties. This is not surprising as the compounds, different to simple hydrocarbons, comprise various functional groups for which biodegradation is extremely difficult to predict.

### 3.5 Peak pattern of non-target peaks at Ergolz site

**Fig. 3:**
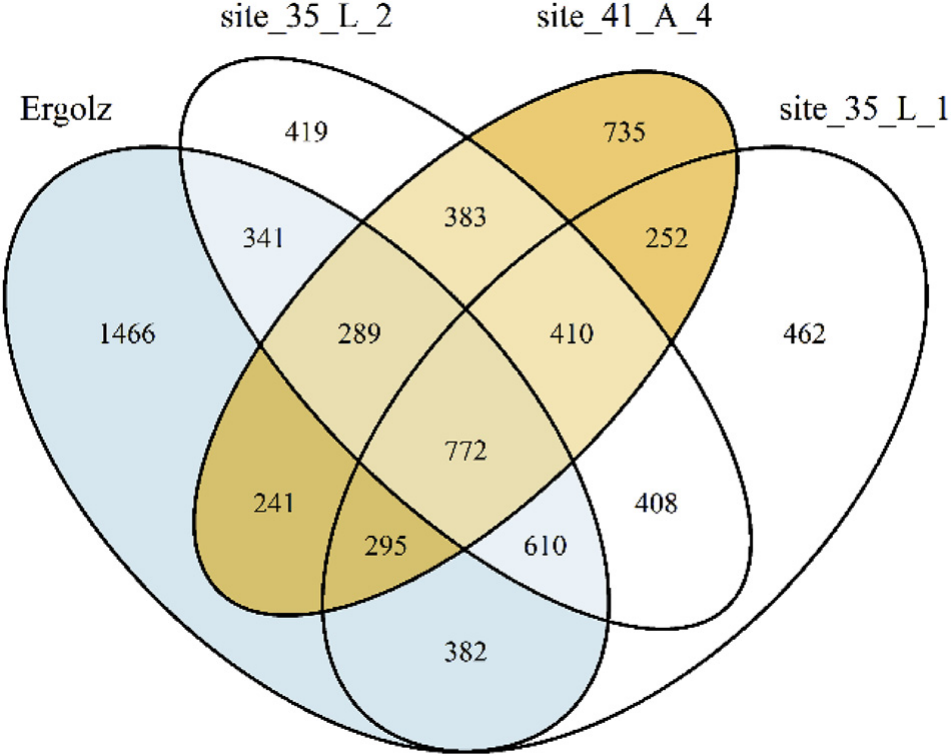
Venn diagram of non-target profile component numbers occurring at the Ergolz sampling sites. Only blind-filtered non-target profiles occurring in all three replicates are included in the diagram. Blue, 24 h-composite sample Ergolz; white, observation well close to the Ergolz (water age. 14 and 39 h, Table 1); brown, drinking water well (water age > 700 h). (For interpretation of the references to colour in this figure legend, the reader is referred to the Web version of this article.)

In addition to the above target screening (see Fig. S5 for concentration distribution for substance classes), a non-target screening using LC-HRMS/MS data was conducted at the Ergolz site to search for further important contaminants in the groundwater used for drinking water production. First, all adduct and isotopologue peaks of target and internal standard compounds as well as peaks occurring in the blank measurements and finally all peaks not measurable in all replicates were removed. Transect profiles were then built for the remaining peaks in the four samples and afterwards combined to component profiles by grouping adduct and isotopologue profiles. Each component is then solely represented by the most intense profile. Altogether 7500 profile components resulted, as depicted in the Venn diagram of Fig. 3. As expected, the highest number of such components was detected in the surface water (4400) whereas the different groundwater samples showed a similar number of profiles (3300–3600). About 800 components can be found in all samples indicating some persistence. The two young groundwater samples at the Ergolz site (35.L.1 and 35.L.2, Fig. 1) have about two-thirds of such non-target components in common, whereas the raw water at extraction well 41.A.4 (around 1.5 km further downstream, Fig. 1) is more different. This finding may be explained by the longer mean travel time and the inflow of regional groundwater from the urbanized part of the catchment or formation of transformation products. This groundwater transports 700 components which can only be found in this latter raw water.

**Fig. 4:**
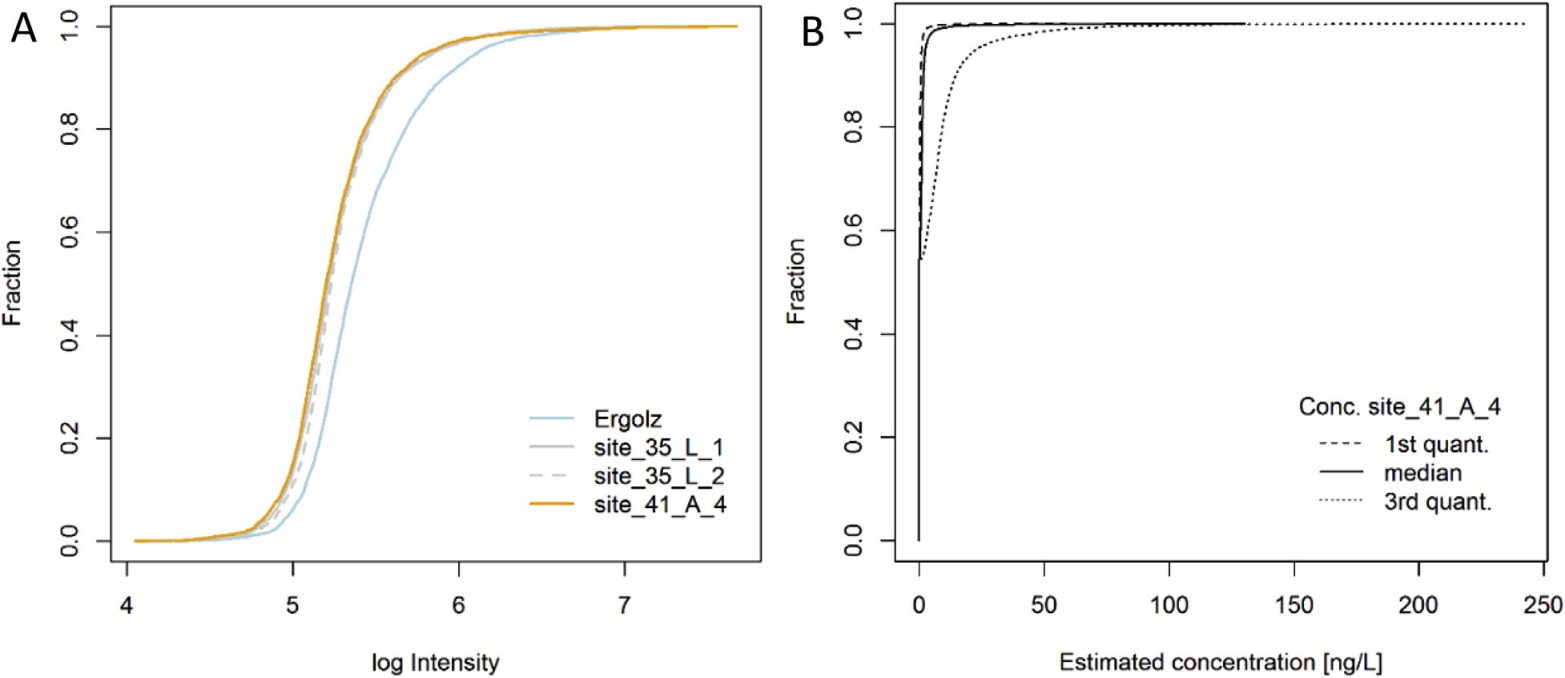
A) Cumulative intensity distribution of the four sampling sites at Ergolz over the total of 7465 component profiles shown in Fig. 3, using the maximum peak intensity per replicate and site for each profile. B) Cumulative distribution of estimated concentrations for the component profiles at the drinking water extraction site. The three quantiles refer to the concentration estimates of each non-target profile formed over the different calibration models utilized for the quantification estimation.

In order to assess the importance of all these non-target components, the cumulative signal intensity is plotted in Fig. 4A. Herein, the intensity drops markedly from the Ergolz river water to the three groundwater samples. For the raw drinking water the concentrations for non-target profiles were estimated using calibration models of 43 randomly selected target-internal standard pairs (Fig. 4B). Thereby, we tried to account for the response fluctuation in ESI-MS by orders of magnitude. Assuming even a relatively low response of the non-target peaks (3rd quantile, dotted line in Fig. 4B), no profile intensities suggested an estimated concentration above 0.1 μg L^−1^ and less than 3% of the profiles might have a concentration between 0.05 and 0.1 μg L^−1^. Therefore, despite the multitude of profiles and their underlying organic compounds detected by the sensitive LC-ESI-HRMS/MS method, no substantial contamination with other polar compounds is expected and consequently no further efforts of profile identification was conducted. It has to be noted though, that such non-target concentration estimates are subject to larger uncertainties than those for target analysis and somewhat depend on the set of calibration models used for their estimation. In addition, very small ionic compounds including certain PMOCs would have to be covered by other enrichment and separation methods (Reemtsma et al., 2016).

### 3.6 Potential implications to human health

The Drinking Water Directive 98/83/EC (1998). Article 4.1 defines that ‘Water intended for human consumption shall be wholesome and clean if it is free […] from substances which, in numbers or concentrations, constitute a potential danger to human health’. Complete absence of any trace contaminant in treated drinking water produced from alluvial aquifers in urbanized river valleys is an illusion, since current analytical techniques as used in this study are capable of detecting very low concentrations. As most of the substances detected have not yet been regulated and lack sufficient toxicity data to derive safe levels, other approaches have to be used to derive threshold values. Often the Threshold of Toxicological Concern (TTC) approach is used to assess potential human health concerns for a chemical based on its structural chemical characteristics and estimated exposure. In Switzerland for perfluorinated surfactants the TTC approach resulted for perfluorooctansulfonic acid and perfluorohexansulfonic acid at 0.3 μg L^−1^, and for perfluorooctanic acid at 0.5 μg L^−1^ (Federal Food Safety and Veterinary Office). Mons et al. (2013) set target values for individual genotoxic and steroid endocrine chemicals at 0.01 μg L^−1^ and for all other organic chemicals at 0.1 μg L^−1^ based on the TTC and further considerations. They addressed also the occurrence of mixtures and proposed target values for the total sum of genotoxic chemicals, the total sum of steroid hormones and the total sum of all other organic compounds at 0.01, 0.01 and 1 μg L^−1^, respectively.

Furthermore, for several individual chemicals the German Federal Office for the Environment has proposed threshold and guiding values (Dieter, 2011). For 1H-benzotriazole, carbamazepine, clofibrate, x-ray contrast media, und diclofenac a precautionary value of 0.1 μg L^−1^ in drinking water was defined.

Unless toxicological studies call for an even lower value (e.g., genotoxic compounds) a threshold value of 0.1 μg L^−1^ for anthropogenic non-natural compounds such as pesticides and pharmaceuticals was also proposed by the International Associations of Water Works of the large rivers in Europe (e.g., Rhine, Danube, Elbe) in their memorandum to protect the provision of drinking water (IAWR, 2013). For all other compounds without known effects they proposed 1 μg L^−1^.

The widely suggested threshold value of 0.1 μg L^−1^ was exceeded in the raw drinking water analysed in this study under dry weather conditions for only four out of the more than 500 compounds analysed. Acesulfam exceeded the threshold value significantly at all sites, showing the high usage of this artificial sweetener. The other sweetener sucralose exceeded the threshold value only slightly at one site (0.12 μg L^−1^). Otherwise, benzotriazole (0.1 μg L^−1^) and tris(2-cloropropylphosphate (flame retardant, 0.12 μg L^−1^) showed each an exceedance at one of the sites. The total concentration of all compounds is at two of the sites above the proposed threshold of 1 μg L^−1^ by Mons et al. (2013), however, this is not surprising when so many compounds are analysed. Overall, the risk associated with the target and the detected non-target compounds of the raw water at the three sites seems relatively low. However, especially at Frenke and Birs the short residence times lead to vulnerability with regard to contamination as the treatment of the raw water is only minor (UV disinfection) and most probably will not reduce the concentration of MPs significantly. In case of rain events untreated wastewater and during pesticide application periods pesticides might reach the surface water in high concentrations, thereby leading to elevated concentrations also in the raw water after short timeframes. Additionally, accidental spills from industry or other sources as detected even in the Rhine river with high dilution (Hollender et al., 2017) cannot be ruled out. Therefore, the anthropogenic activities in the catchment such as wastewater treatment and industrial production should be monitored and the raw water quality regularly investigated. An additional barrier such as activated carbon or sand filtration to eliminate accidental spills could be beneficial.

## 4 Conclusions

Multi-target and non-target screening of MPs at three RBF sites revealed that RBF is an effective natural process to reduce or even remove MPs from river water, and even for the short residence times observed.

• From the 461 MPs analysed with sufficient sensitivity, 75–96 MPs were detected in the rivers and therefrom 43–59% eliminated during RBF.
• No correlation of natural attenuation of MPs with physico-chemical properties was revealed.
• The remaining total concentrations of the MPs in the raw drinking water accounted to 0.6–1.6 μg L^−1^.
• During the sampling period only a few of the targeted compounds exceeded 0.1 μg L^−1^, a threshold value often used to indicate the absence of health risks.
• Non-target screening at one site indicates no further high contamination with organic contaminants during the sampling period.

## Conflict of Interest statement

The authors report no conflict of interest.
