# Supplementary material for: Comprehensive micropollutant screening using LC-HRMS/MS at three riverbank filtration sites to assess natural attenuation and potential implications for human health
Source: Water Res X. 2018 Nov 2;1:100007. doi: 10.1016/j.wroa.2018.100007 (PMC6549901; doi:10.1016/j.wroa.2018.100007)
Supplement: Multimedia component 1 [file mmc1.pdf]

SUPPORTING INFORMATION TO ARTICLE:

Comprehensive micropollutant screening using LC-HRMS/MS at three riverbank filtration sites to assess natural attenuation and potential implications for human health

Juliane Hollender[1,3]*, Judith Rothardt[1], Dirk Radny[1], Martin Loos[1], Jannis Epting[2], Peter Huggenberger[2,] Paul Borer[1], Heinz Singer[1]

[1]Eawag: Swiss Federal Institute of Aquatic Science and Technology, Ueberlandstrasse 133, 8600 Duebendorf, Switzerland,

[2]Applied and Environmental Geology, University of Basel, Bernoullistrasse 32, 4056 Basel, Switzerland

[3]Institute of Biogeochemistry and Pollutant Dynamics, Universitätstrasse 16, ETH Zürich, 8092 Zürich, Switzerland

* Corresponding author

List of content:

SI 1 Material and methods **2**

Table S1. Parameters used for peak picking 2

Table S2. Parameters used for the ISTD-based recalibration 2

Table S3. Parameters used in the screening of target and ISTD compounds 3

Table S4. Parameters used for blank- and blind-peak subtraction 3

Table S5. Parameters used for non-target grouping 3

Table S6. Parameters used for the extraction of site-intensity profiles 3

SI 2 Results and discussion **3**

Table S7. Limits of quantification (LOQ), and concentrations of all detected MPs [ng/L] 3

Table S8: Prediction of biodegradability 4

Fig. S1. Number and distribution of the target compounds to the different substance classes 5

Fig. S2. LogD values of all compounds for pH 6.6-6.8 predicted by Episuite 5

Fig. S3: Relative recoveries for MPs. 6

Fig. S4: Limits of quantification (LOQs) for MPs 6

Fig. S5: Boxplots of the concentrations at the Ergolz site 7

SI 1 Material and methods

Determination of organic carbon content

The fraction of organic carbon (f_{OC}) of the aquifer material was measured from sediment cores that were taken in the vicinity of the investigated transects at two of the three sites (Frenke and Ergolz) by using a Geoprobe® Direct Push drilling rig and the Macro-Core® soil sampler. Aquifer samples at different depths (1-10 m) were dried and sieved. Subsequently, the fine material < 0.2 mm was ground and analyzed for the organic carbon content using an elemental Analyzer (EA3000, Eurovector) and subtracting the inorganic carbon content determined by acidification and coulometric detection on a CO2- Coulometer (CM515, UIC inc.), both in triplicates. The organic carbon content in the fine material of the aquifer material at the sites of Frenke and Ergolz ranged from 0.1 to 1%. With a typical particle size contribution of the fine material < 0.2 mm of 2-5% determined for the Frenke sample, the f_{OC} of the whole aquifer material at the investigated sites can be assumed to range in between 0.002% and 0.05%.

Characterisation of unknown peaks

Table S1. Parameters used for peak picking with the R *enviPick* package, functions *mzagglom()*, *mzclust()* and *mzpick()*, respectively. Check the package manual for further parameter descriptions.

Parameter	Value
dmzgap	*>3.5*
ppm	*TRUE*
drtgap	*300 [seconds]*
minpeak	*4*
maxint	*$1x10^7$*

Parameter	Value
dmzdens	*3.5*
ppm	*TRUE*
drtdens	*60 [seconds]*
minpeak	*4*
maxint	*$1x10^7$*

Parameter	Value
minpeak	*4*
drtsmall	*20 [seconds]*
drtfill	*10 [seconds]*
drttotal	*120 [seconds]*
recurs	*2*
weight	*1*
SB	*4*
SN	*5*
minint	*$1x10^4$*
maxint	*$1\ x10^7$*
ended	*1*

Table S2. Parameters used for the ISTD-based recalibration of m/z peak values (*enviMass* interface settings).

Parameter	Value
m/z tolerance	*3 ppm*
RT tolerance	*30 [seconds]*

Table S3. Parameters used in the screening of target and ISTD compounds (*enviMass* interface settings).

Parameter	Value
RT tolerance of peaks relative to their expected RT [s]	*60 [seconds]*
RT tolerance of peaks within an isotope pattern [s]	*15 [seconds]*
m/z tolerance	*3 ppm*
Intensity tolerance	*30 %*
Cutoff score	*0.8*

Table S4. Parameters used for blank- and blind-peak subtraction (*enviMass* interface settings).

Parameter	Value
Intensity threshold ratio sample/blind	*100*
m/z tolerance	*3 ppm*
RT tolerance	*30 [seconds]*

Table S5. Parameters used for non-target grouping (*enviMass* interface settings).

Parameter	Value
EIC correlation, RT tolerance	*5 [seconds]*
EIC correlation, minimum number of centroids per EIC	*10*
EIC correlation, Pearson correlation threshold	*0.95*
Isotopologue grouping, m/z tolerance	*2.5 ppm*
Isotopologue grouping, RT tolerance	*5 [seconds]*
Isotopologue grouping, intensity tolerance	*50 %*
Adduct grouping, m/z tolerance	*2.5 ppm*
Adduct grouping, RT tolerance	*5 [seconds]*
Adduct grouping, included adducts	*[$M+H^+$, $M+Na^+$, $M+K^+$, $M+NH4^+$]*

Table S6. Parameters used for the extraction of site-intensity profiles (*enviMass* interface settings).

Parameter	Value
m/z tolerance	*3 ppm*
RT tolerance	*60 [seconds]*

SI 2 Results and discussion

Table S7. Limits of quantification (LOQ), and concentrations of all detected MPs [ng/L] in river water, abstraction, and extraction well samples collected in December 2013. Compounds not detected in recovery samples were annotated by “nd”. No entry means that the compounds is not detected in the environmental sample, # interference. Compounds written in italic were quantified via an isotope labelled internal standard. The table is enclosed as excel file.

Table S8. Prediction of biodegradability using the six aerobic and one anaerobic models which correlate the presence of structural fragments with biodegradation data from different databases (BIOWIN, US EPA; Howard et al. 1992, Jaworska et al. 2003)

Compound	CAS-Nr	SMILE	logP	$logK_{OW}$	BioWin 1	BioWin 2	BioWin 3	BioWin 4	BioWin 5	BioWin 6	BioWin 7 (anaerobic)	Ready Biodegra-dability	Speciation @pH 7	pKa
**Natural attenuated**														
4-Acetamidoantipyrin (4-AAA)	83-15-8	CC(=O)NC=2C(=O)N(c1ccccc1)N(C)C=2C	0.15	0.3	0.97	0.98	2.62	3.7	0.15	0.05	-0.23	NO	n	12.52
Atenolol	29122-68-7	CC(C)NCC(O)COc1ccc(cc1)CC(N)=O	0.43	0.16	1.33	1	2.61	3.85	0.41	0.23	-0.19	NO	c	9.67
Bezafibrate	41859-67-0	c1(C(NCCc2ccc(OC(C(O)=O)(C)C)cc2)=O)ccc(Cl)cc1	3.99	4.25	0.68	0.57	2.16	3.61	0.27	0.04	-1.12	NO	a	3.83
Caffeine	58-08-2	c12c(n(c(=O)n(c1=O)C)C)ncn2C	-0.55	-0.07	0.66	0.56	2.77	3.57	0.14	0.05	0.5	NO	n	0.92
Clopidogrel carboxylic acid	144457-28-3	Clc1ccccc1C(N3Cc2c(scc2)CC3)C(=O)O	1.23	1.51	0.34	0.01	2.35	3.27	-0.15	0.005	-1.24	NO	z	7.9
Diclofenac	15307-86-5	c1c(c(ccc1)Nc1c(cccc1Cl)Cl)CC(=O)O	4.26	4.02	0.14	0.003	2.29	3.3	-0.13	0.003	-0.85	NO	a	4.00
Etodolac	41340-25-4	O=C(O)CC3(OCCc2c3nc1c(cccc12)CC)CC	3.44	3.93	0.26	0.01	2.56	3.52	0.14	0.01	-0.33	NO	a	4.73
Flufenamic acid	530-78-9	FC(F)(F)c1cc(ccc1)Nc2ccccc2C(=O)O	5.25	5.25	0.04	0.002	2.02	3.07	0.21	0	0.06	NO	a	3.88
Irbesartan	138402-11-6	C12(C(N(Cc3ccc(c4c(c5[nH]nnn5)cccc4)cc3)C(=N1)CCCC)=O)CCCC2	5.5	5.31	0.68	0.43	2.28	3.55	-0.13	0.004	-1.64	NO	a	4.12
Metformine	657-24-9	CN(C)C(=N)NC(N)=N	-0.92	-2.64	0.69	0.76	2.91	3.66	0.33	0.24	0.68	NO	c	10.27
Metoprolol	37350-58-6	COCCC1=CC=C(OCC(O)CNC(C)C)C=C1	1.76	1.88	0.77	0.7	2.65	3.64	0.33	0.15	0.07	NO	c	9.67
Naproxen	22204-53-1	c12c(cc(OC)cc2)ccc(c1)[C@@H](C(O)=O)C	2.99	3.18	0.9	0.96	2.92	3.91	0.44	0.35	0.39	NO	a	4.19
Saccharine	81-07-2	c12c(C(=O)NS1(=O)=O)cccc2	0.45	0.91	0.66	0.6	2.79	3.58	0.2	0.09	0.45	NO	a	2.84
Sitagliptin	486460-32-6	Fc1cc(c(F)cc1F)C[C@@H](N)CC(=O)N3Cc2nnc(n2CC3)C(F)(F)F	1.26	1.39	-1.98	0	0.46	3.21	-0.29	0	-0.25	NO	c	8.78
Trimethoprim	738-70-5	c1(Cc2c(nc(N)nc2)N)cc(c(OC)c(c1)OC)OC	1.28	0.73	0.59	0.92	2.04	3.37	0.09	0.02	0.17	NO	c	7.16
Valsartan	137862-53-4	CCCCC(=O)N(CC1=CC=C(C=C1)C2=C(C=CC=C2)C3=NN=NN3)[C@@H](C(C)C)C(O)=O	5.27	3.65	0.93	0.88	2.85	4.08	-0.22	0.002	-1.13	NO	a	4.37
Valsartanic acid	164265-78-5	O=C(O)c1ccc(cc1)c2ccccc2c3nnnn3	3.18	1.83	0.8	0.84	2.7	3.47	0.36	0.15	0.34	NO	a	4.03
**Persistent**														
Acesulfame	55589-62-3	S1(NC(C=C(O1)C)=O)(=O)=O	-0.55	-1.33	0.67	0.67	2.84	3.61	0.23	0.11	0.68	NO	a	3.02
Atrazine-2-Hydroxy	2163-68-0	c1(nc(nc(n1)O)NCC)NC(C)C	1.66	2.09	0.31	0	2.3	3.33	-0.01	0	-0.1	NO	n	2.99
Candesartan	139481-59-7	c1(ccccc1c1ccc(Cn2c(nc3c2c(C(=O)O)ccc3)OCC)cc1)c1nnn[nH]1	5.17	4.79	0.85	0.81	2.26	3.3	0.06	0.008	0.17	NO	a	3.93
Carbamazepine	298-46-4	N1(c2c(cccc2)C=Cc2c1cccc2)C(N)=O	2.77	2.45	0.64	0.41	2.68	3.51	0.09	0.04	-0.07	NO	n	15.96
Chloridazon-desphenyl	6339-19-1	c1(c(nncc1N)O)Cl	-0.78	-0.41	0.38	0.11	2.59	3.4	0.2	0.08	0.14	NO	z	6.63
Chloridazon-methyl-desphenyl	17254-80-7	ClC(=C(C=N1)N)C(=O)N1C	-0.55	-1.37	0.71	0.5	2.7	3.56	0.28	0.04	0.96	NO	n	15.79
2-6-Dichlorbenzamide	2008-58-4	ClC1=C(C(N)=O)C(Cl)=CC=C1	2.03	0.9	0.5	0.26	2.31	3.44	0.31	0.1	-0.82	NO	n	12.08
Hydrochlorothiazide	58-93-5	NS(=O)(=O)C1=CC2=C(NCNS2(=O)=O)C=C1Cl	-0.58	-0.07	0.19	0.006	2.2	3.14	-0.29	0	-0.15	NO	n	9.09
Lamotrigine	84057-84-1	Cl-c(ccc1)c(Cl)c1-c(nnc2N)c(n2)N	1.93	2.57	-0.21	0	1.95	2.93	-0.33	0	-0.81	NO	n	5.87
5-Methyl-Benzotriazole	136-85-6	c12c(cc(C)cc2)nn[nH]1	1.81	1.71	0.74	0.85	2.83	3.59	0.38	0.34	0.18	NO	n	8.86
Metolachlor-ESA	171118-09-5	O=S(O)(CC(N(C(C)COC)C1=C(CC)C=CC=C1C)=O)=O	2.11	1.69	0.67	1	2.45	3.61	-0.08	0.008	-1.12	NO	a	13.72
Sucralose	56038-13-2	OC1C(OC(CO)C(Cl)C1O)OC2(CCl)OC(CCl)C(O)C2O	-0.47	-1	-0.21	0	2.36	3.44	0.66	0.004	0.74	NO	n	11.91
Sulfamethoxazole	723-46-6	c1(S(Nc2cc(C)on2)(=O)=O)ccc(N)cc1	0.79	0.89	0.45	0.13	2.43	3.51	-0.12	0.006	-0.29	NO	n	1.97

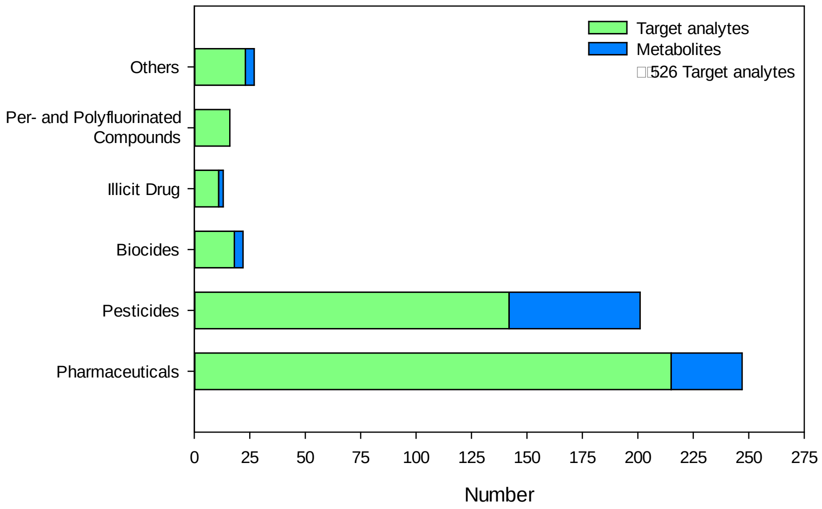

Fig. S1. Number and distribution of the target compounds (Table S7) to the different substance classes

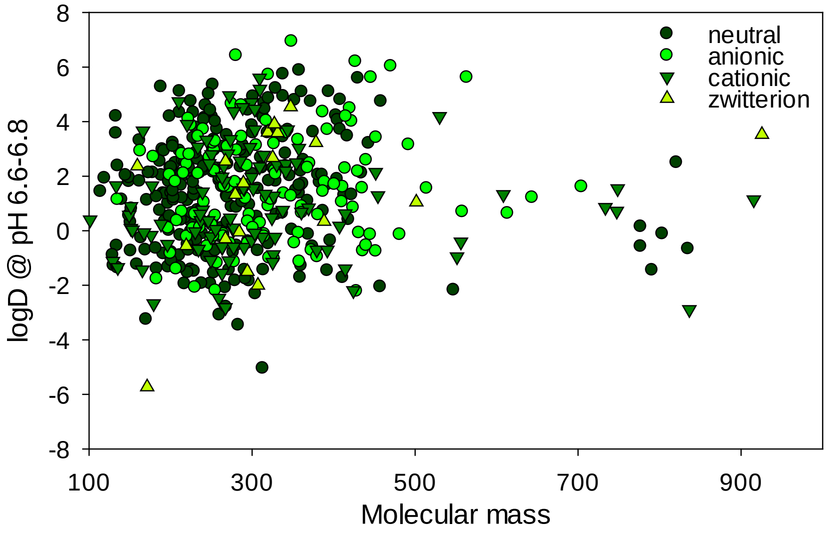

Fig. S2. LogD values of all compounds (Table S7) calculated for pH 6.6-6.8 predicted by Episuite (US EPA, 2013)

Reference
US EPA (2012) Estimation Programs Interface Suite™ for Microsoft Windows, v. 4.11; 2012, United States Environmental Protection Agency, Washington, DC, USA.

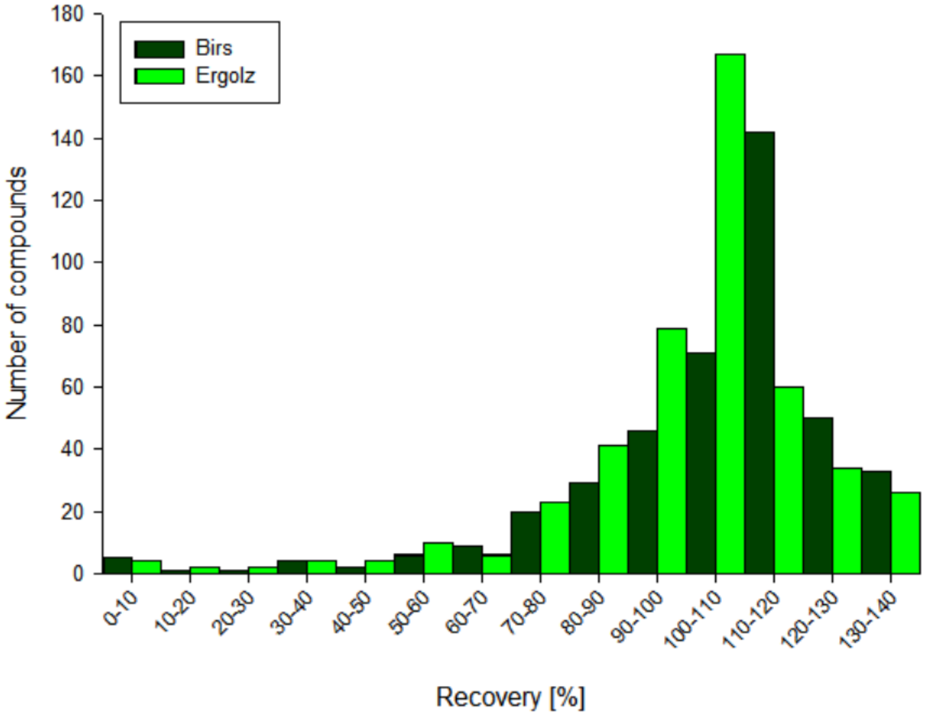

Fig. S3: Relative recoveries for MPs determined for the 24h composite sample from the Birs river water as well as the Ergolz abstraction well sample 41.A.4. Recoveries were similar for both matrices.

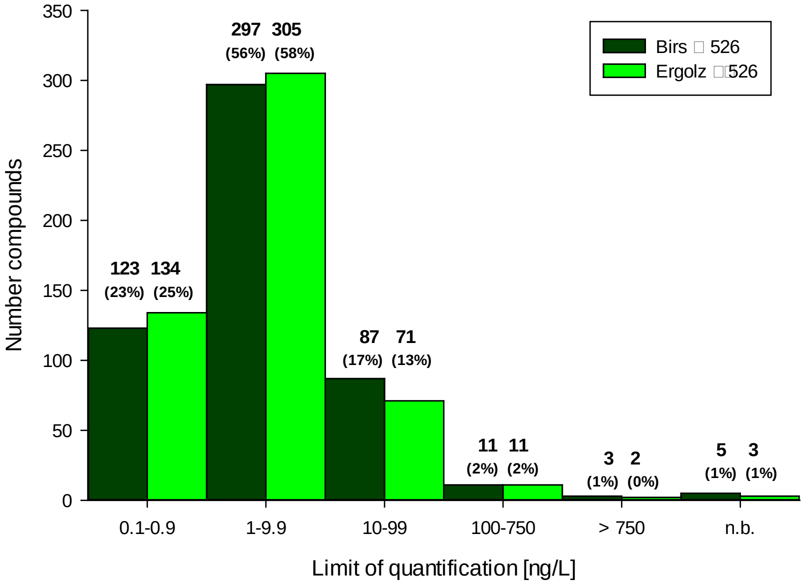

Fig. S4: Limits of quantification (LOQs) for MPs determined for the 24h composite sample from the Birs river water as well as the Ergolz abstraction well sample 41.A.4. For 65 analytes of 526 compounds no LOQ could be determined at all, due to values above the highest calibration point of 750 ng/L or matrix interferences. For individual values see Table S7.

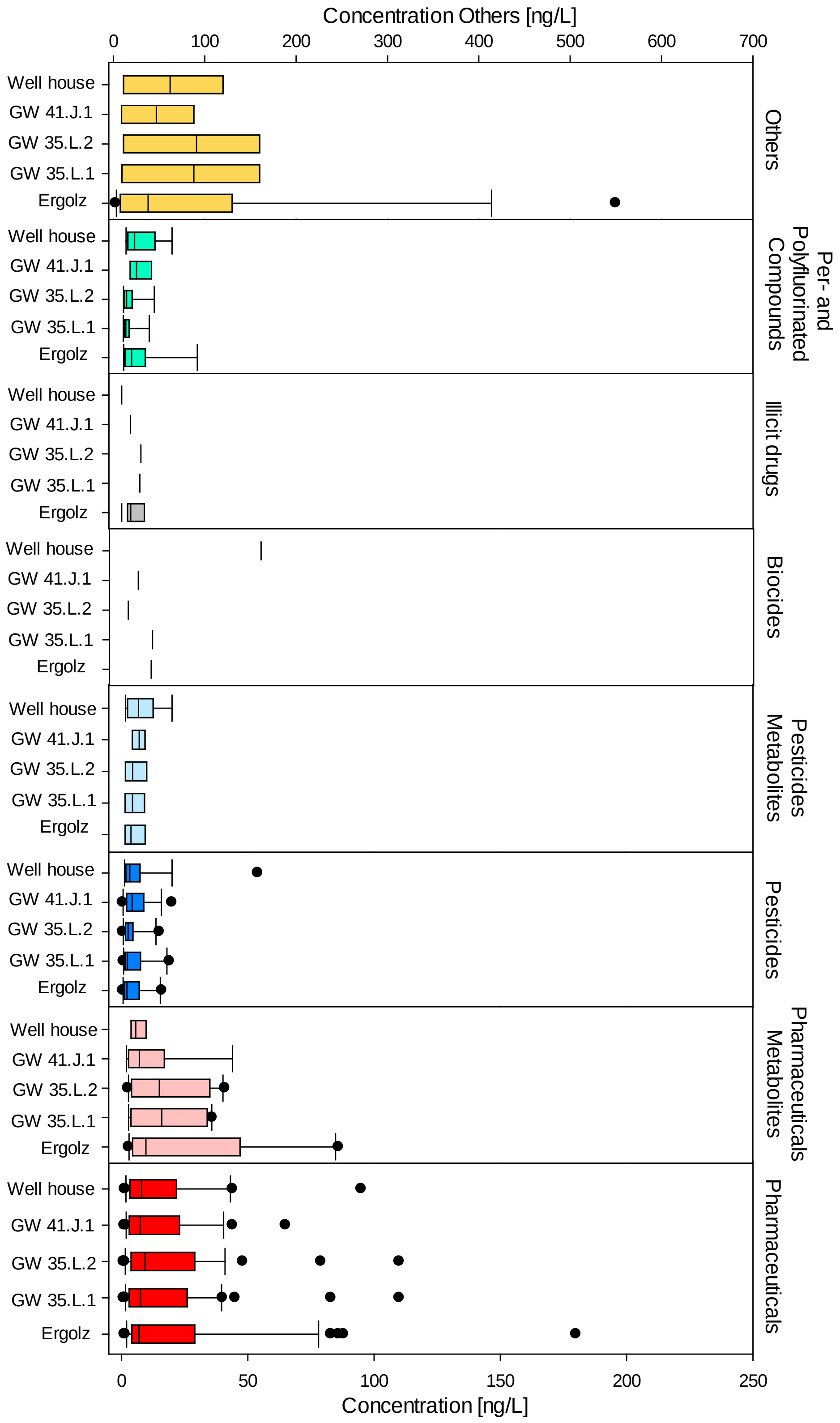

Fig. S5: Boxplots of the concentrations with median, the first and third quartile, whiskers representing the standard deviations, and outliers for the different substance classes at the Ergolz site.
